# Land cover maps of Antananarivo (capital of Madagascar) produced by processing multisource satellite imagery and geospatial reference data

**DOI:** 10.1016/j.dib.2020.105952

**Published:** 2020-06-30

**Authors:** Dupuy Stéphane, Defrise Laurence, Gaetano Raffaele, Andriamanga Valérie, Rasoamalala Eloise

**Affiliations:** a CIRAD, UMR TETIS, F-97410 Saint-Pierre, Réunion, France; b CIRAD, UMR TETIS, 101, Antananarivo, Madagascar; c CIRAD, UMR TETIS, F-34398, Montpellier, France; d TETIS, AgroParisTech, CIRAD, CNRS, INRAE, Univ Montpellier, Montpellier, France

**Keywords:** Remote sensing, Land cover map, Spatial database, Landsat-8, Sentinel-2, Pleiades, OBIA, Antananarivo

## Abstract

We describe a reference spatial database and four land use maps of Antananarivo city produced over 2017 reference year using a methodology combining machine learning and object based image analysis (OBIA). These maps are produced by processing satellite images using the Moringa land cover processing chain developed in our laboratory. We use a single very high spatial resolution (VHSR) Pleiades image, a time series of Sentinel-2 and Landsat-8 images, a Digital Terrain Model (DTM) and the aforementioned reference database. According to the Moringa workflow, the Pleiades image is used to generate a suitable object layer at VHSR using a segmentation algorithm. Each object is then classified using variables from the time series and information from the DTM. The reference database used to train the supervised classification algorithm is here described, as well as the 4 land cover maps produced at four different hierarchically nested nomenclature levels. For a number of classes going from 2 to 20, overall accuracies range from 94% to 74%. Such land cover products are very rare in Madagascar, so we have decided to make them openly accessible and usable by land managers and researchers.

Specifications table

**Table:** 

Subject	Computer Science, Earth Sciences, Social Sciences
Specific subject area	Remote Sensing, GIS, Land Cover Map
Type of data	Vector
How data were acquired	The reference database was created with the QGIS software (www.qgis.org) For the production of land use maps, the Moringa processing chain uses the Orfeo ToolBox software (www.orfeo-toolbox.org) driven by Python scripts. The source code of the Moringa processing chain is available at https://gitlab.irstea.fr/raffaele.gaetano/moringa.git
Data format	Raw data (Shapefile, Esri)
Parameters for data collection	To build the reference database, plots were chosen in order to have (i) a good representativeness of each class and (ii) a homogeneous distribution of classes over the study area
Description of data collection	To build the reference database, GPS waypoints were collected during the end of 2017 rainy season. A Trimble Yuma2 tablet was used to collect the waypoints. Each waypoint was then converted into a polygon by digitizing the boundaries of the corresponding land cover using the VHSR Pleiades image as a support for photo-interpretation. To produce the land use maps, the Moringa processing chain was used, implementing aa supervised classification method for satellite images (Sentinel2, Landsat8 and Pleiades) based on the Random Forest algorithm driven by the reference database mentioned above. We produced four land use maps using the reference database and satellite image classifications as described below.
Data source location	Antananarivo, capital of Madagascar located in the Indian Ocean (upper left corner: 18°43′37.71′’S and 47°19′23.42′’E // lower right corner: 19°06′07.73′’S and 47°39′14.21′’E)
Data accessibility	Repository name: CIRAD Dataverse Data identification number: Land Use Map: Dupuy, Stéphane; Defrise, Laurence; Gaetano, Raffaele; Burnod, Perrine, 2019, "Antananarivo - 2017 Land cover map", doi:10.18167/DVN1/NHE34C, CIRAD Dataverse, V2 Reference database: Laurence, Defrise; Andriamanga, Valérie; Rasoamalala, Eloise; Dupuy, Stéphane; Burnod, Perrine, 2019, "Antananarivo - Madagascar - 2017, Land use reference spatial database", doi:10.18167/DVN1/5TZOOW, CIRAD Dataverse, V1 Direct URL to data: Data are referenced in the CIRAD Dataverse and are hosted on CIRAD's Aware Geographic catalog. The web links are in the following files. Land use map: http://dx.doi.org/10.18167/DVN1/NHE34C Reference database: http://dx.doi.org/10.18167/DVN1/5TZOOW
Related research article	S. Dupuy, L. Defrise, V. Lebourgeois, R. Gaetano, P. Burnod, J.-P. Tonneau, analyzing Urban Agriculture's Contribution to a Southern City's Resilience through Land Cover Mapping: The Case of Antananarivo, Capital of Madagascar, Remote Sensing. 12 (2020). https://doi.org/10.3390/rs12121962.

## Value of the data

• The maps can be used by institutions and land planners to update urban and sanitation master plans.
• The reference database can be used by remote sensing specialists to assess new methods for land cover mapping and other classification algorithms.
• All data provided is georeferenced and in vector format for use in GIS tools in future projects.

## 1 Data description

**Fig. 1:**
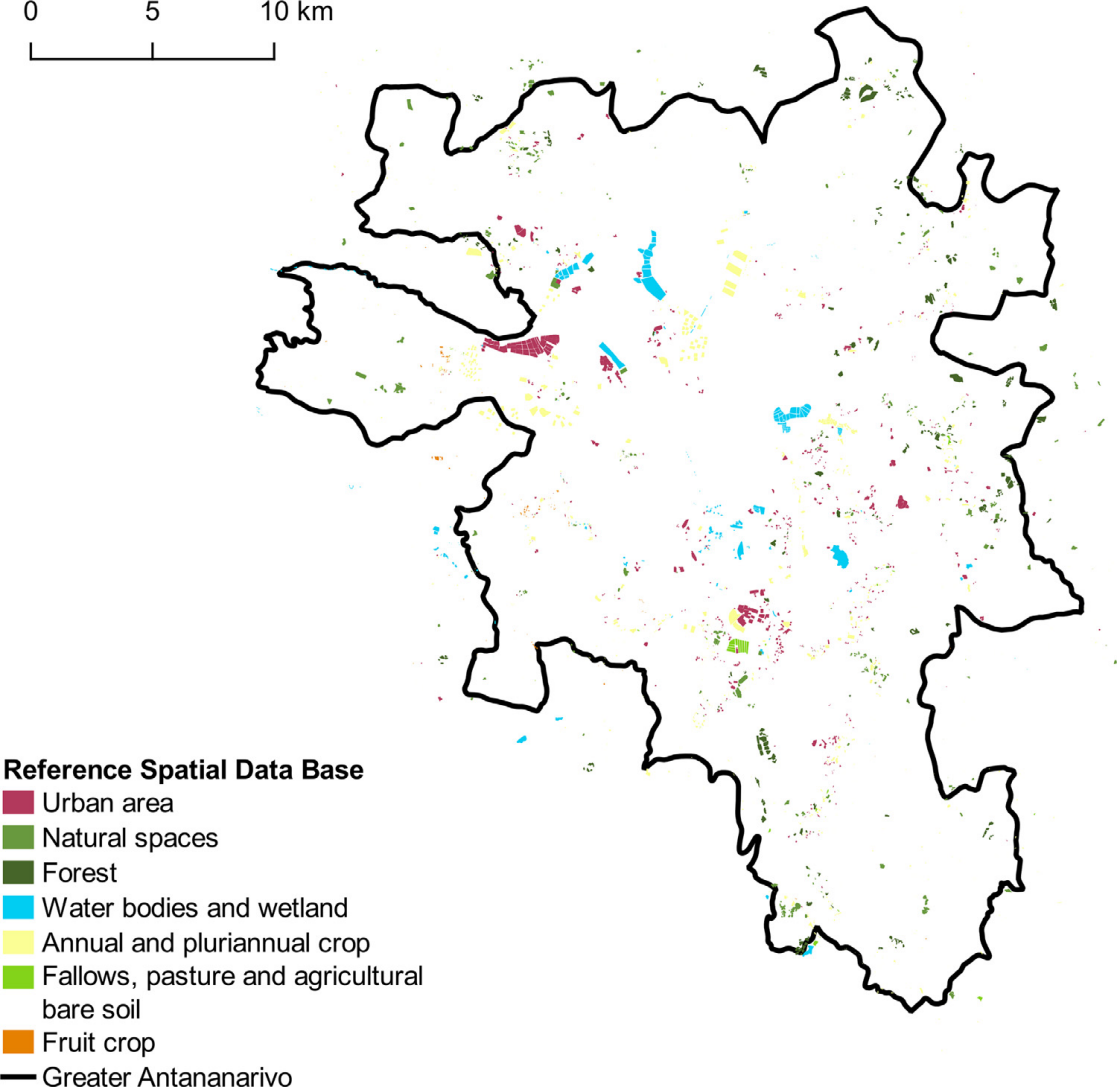
Distribution of the collected polygons - Vector file in ESRI shape format available here: http://dx.doi.org/10.18167/DVN1/5TZOOW.
• *Four land use maps* produced by processing multisource satellite data including a Very High Spatial Resolution (VHRS) Pleiades image, a time series of HRS Sentinel-2 and Landsat-8 images and a digital terrain model. Each map correspond to land use at one of the four levels of nomenclature (from 2 to 20 classes), and is distributed in vector format (shapefile). Each geometry corresponds to an object provided by the segmentation of the Pleiades image, attributed using a class label at the specific nomenclature level. Validation results show that map accuracies range from 76% for the most detailed nomenclature level (20 classes) to 94% for the least detailed level (2 classes). The four maps produced are illustrated in Figs. 2–5.

The data described in this paper are of two different types related to land use on the greater Antananarivo area:

• *A GIS reference database* in ESRI shapefile format composed of 3068 polygons representative of the diversity of land uses in Antananarivo. Each polygon is annotated with four class labels corresponding to four levels of nomenclature. Class nomenclatures are hierarchically nested, and the number of classes ranges from 2 at level 1 (crop vs. non-crop) to 20 at level 4. Detailed hierarchical nomenclature is shown in Table 1. This database is used to generate training samples in the Moringa supervised classification process in order to identify land use classes from a set of variables extracted from high and very high spatial resolution satellite images. We use the reference database to evaluate the accuracy of the provided land use maps with a cross-validation technique. The spatial distribution of reference polygons is depicted in Fig. 1.

**Table 1:** Nomenclature presenting the four levels of precision and the number of polygons in reference database level 4.

Level 1 Crop Land	Level 2 Land Cover	Level 3 Crop Group	Level 4 Crop Class	Number of polygons
Non crop	Urban area	Built-up surface	Mixed habitat	284
			Residential area	149
			Rural housing	110
		Industrial, commercial and military area	Industrial, commercial and military area	99
		Quarry, landfill and construction site	Quarry, landfill and construction site	60
			Brick extraction	111
	Natural spaces	Bare non-agricultural soil	Bare non-agricultural soil	87
		Savannah	Herbaceous savannah	111
			Shrub savannah	155
	Forest	Forest	Tree savannah	196
			Pines	110
	Waterbodies and wetland	Water bodies	Water bodies	143
		Wetland	Wetland	80
Crop	Annual and pluriannual crops	Irrigated crop	Rice	350
			Watercress	83
		Vegetable crop	Vegetable crop	371
		Rainfed crop	Cassava	192
			Other rainfed crop	140
	Fallows, pasture and agricultural bare soil	Fallows, pasture and bare agricultural soil	Fallows, pasture and bare agricultural soil	99
	Fruit crop	Fruit crop	Fruit crop	138
			TOTAL	3 068

**Fig. 2:**
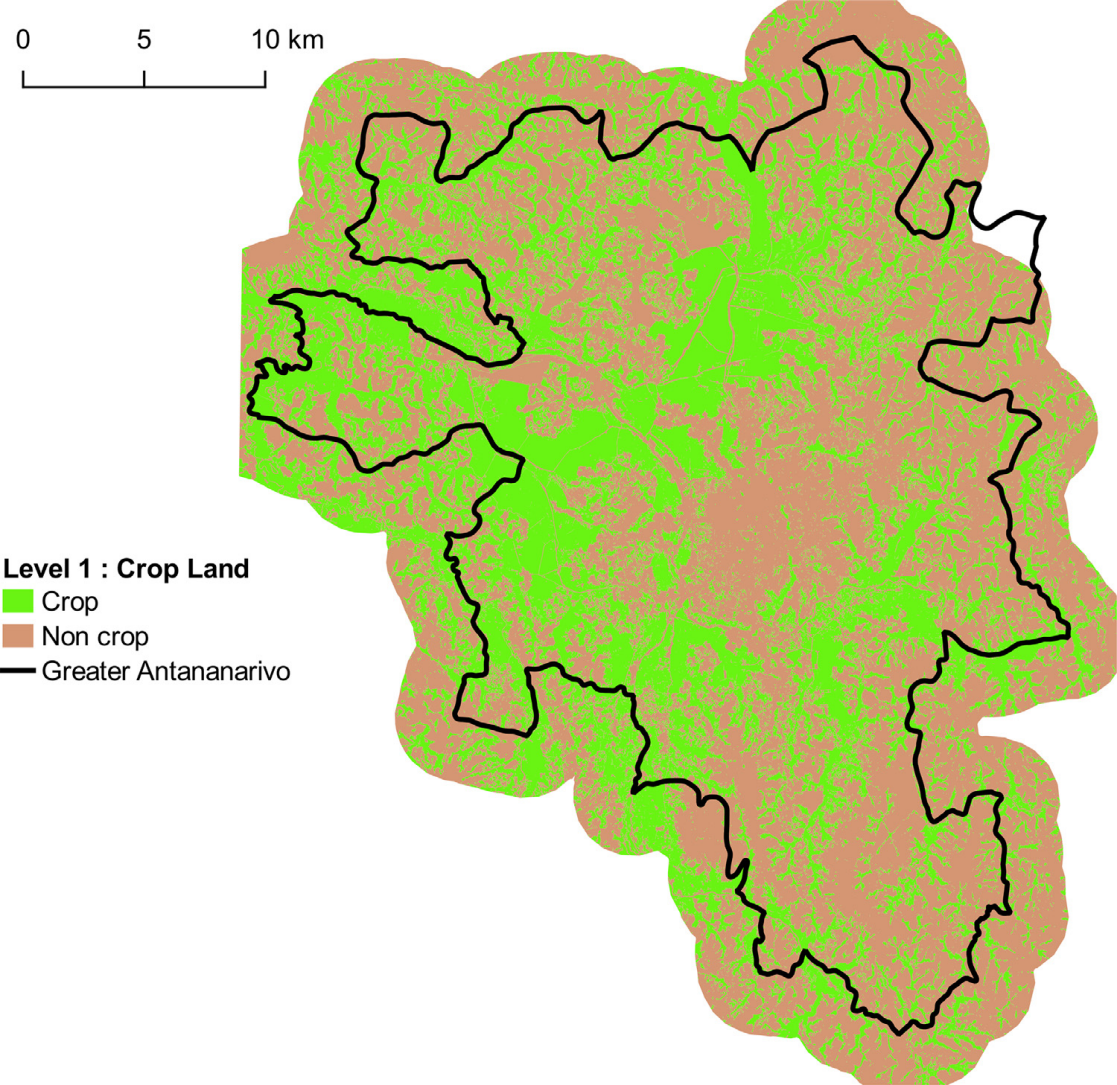
Land cover map corresponding to Level-1 with 2 classes. Vector file in ESRI shape format available here: http://dx.doi.org/10.18167/DVN1/NHE34C. This figure is a modified version of Fig. 5 published in this article [1].

**Fig. 3:**
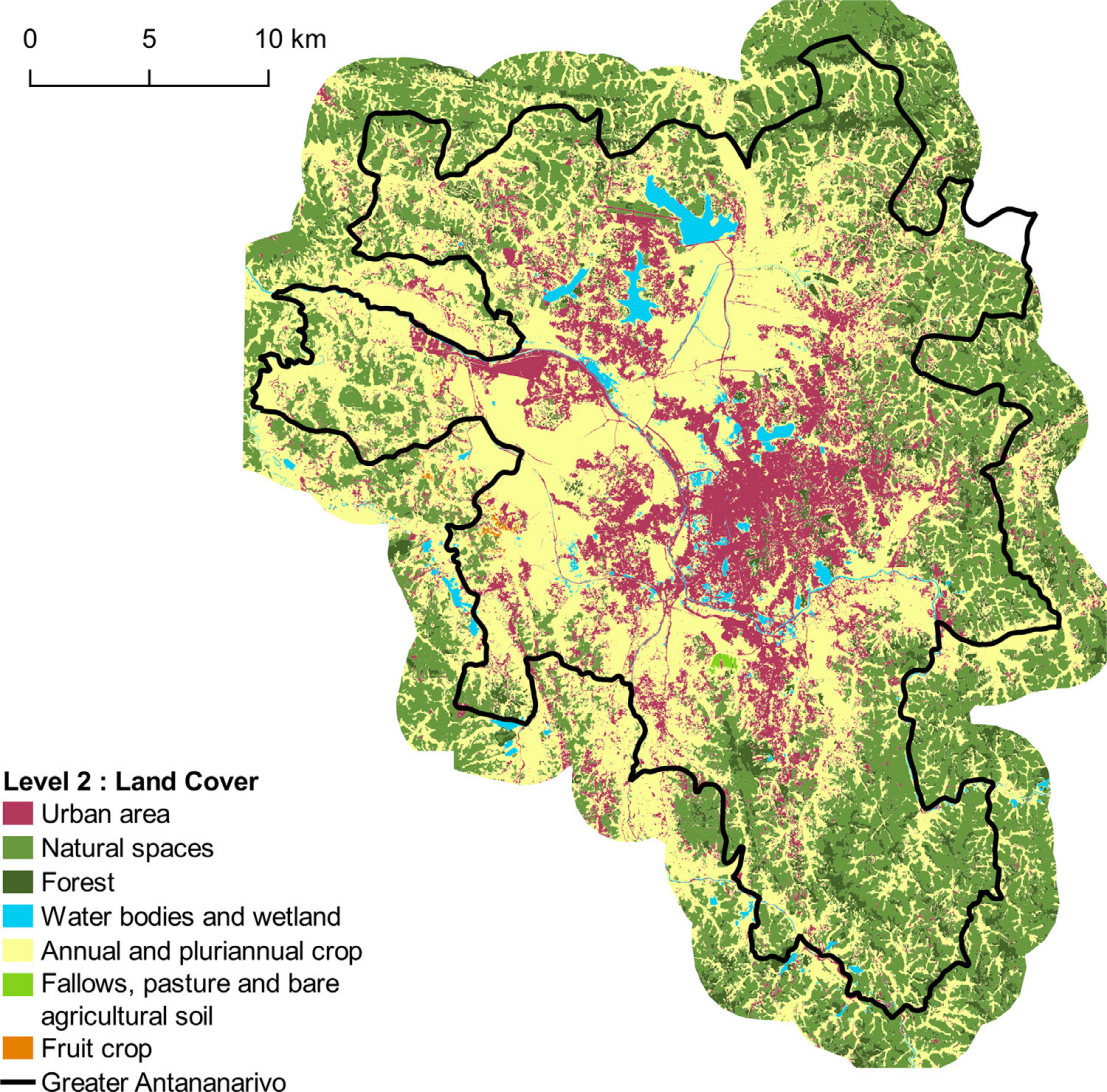
Land cover map corresponding to Level-2 with 8 classes. Vector file in ESRI shape format available here: http://dx.doi.org/10.18167/DVN1/NHE34C. This figure is a modified version of Fig. 5 published in this article [1].

**Fig. 4:**
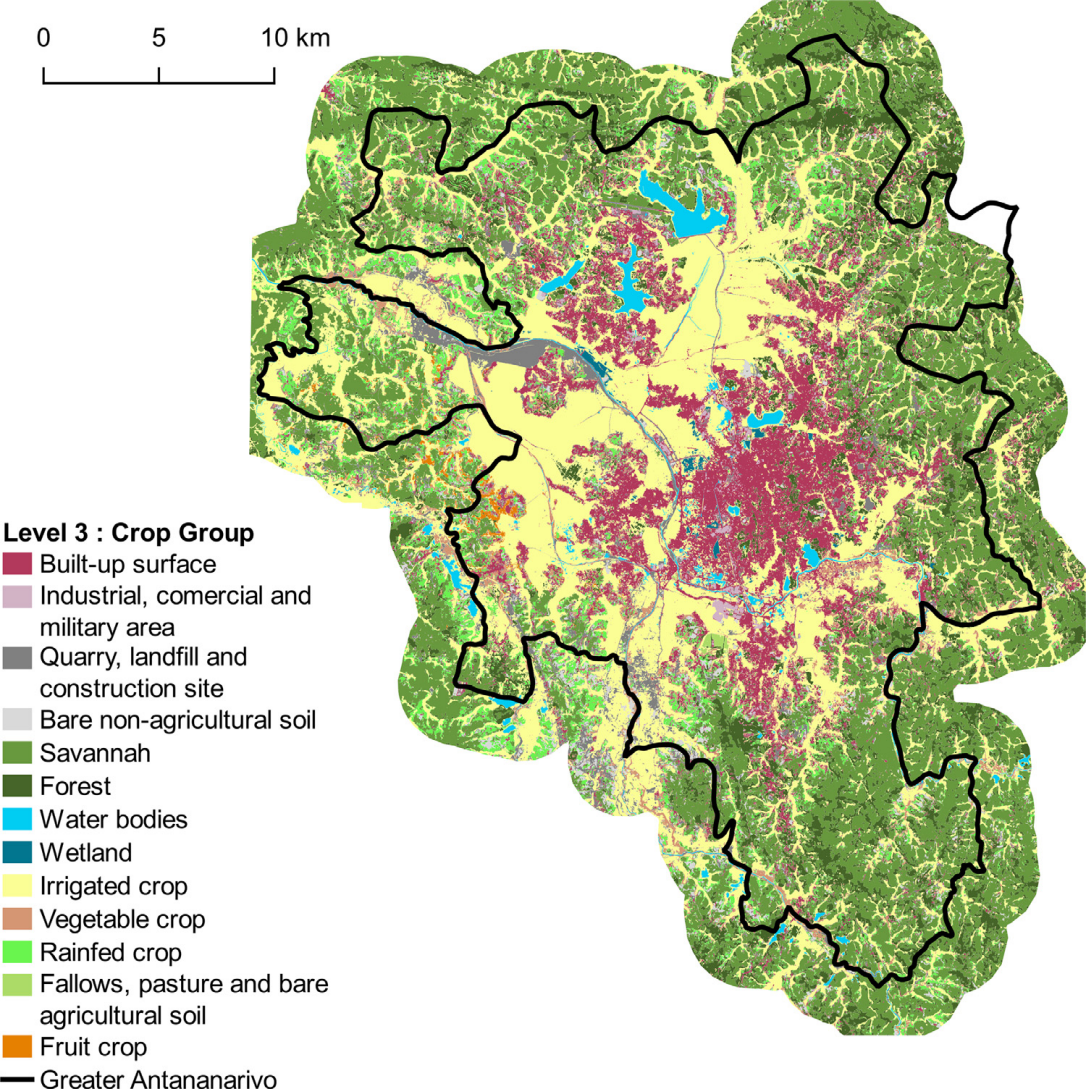
Land cover map corresponding to Level-3 with 13 classes. Vector file in ESRI shape format available here: http://dx.doi.org/10.18167/DVN1/NHE34C.

**Fig. 5:**
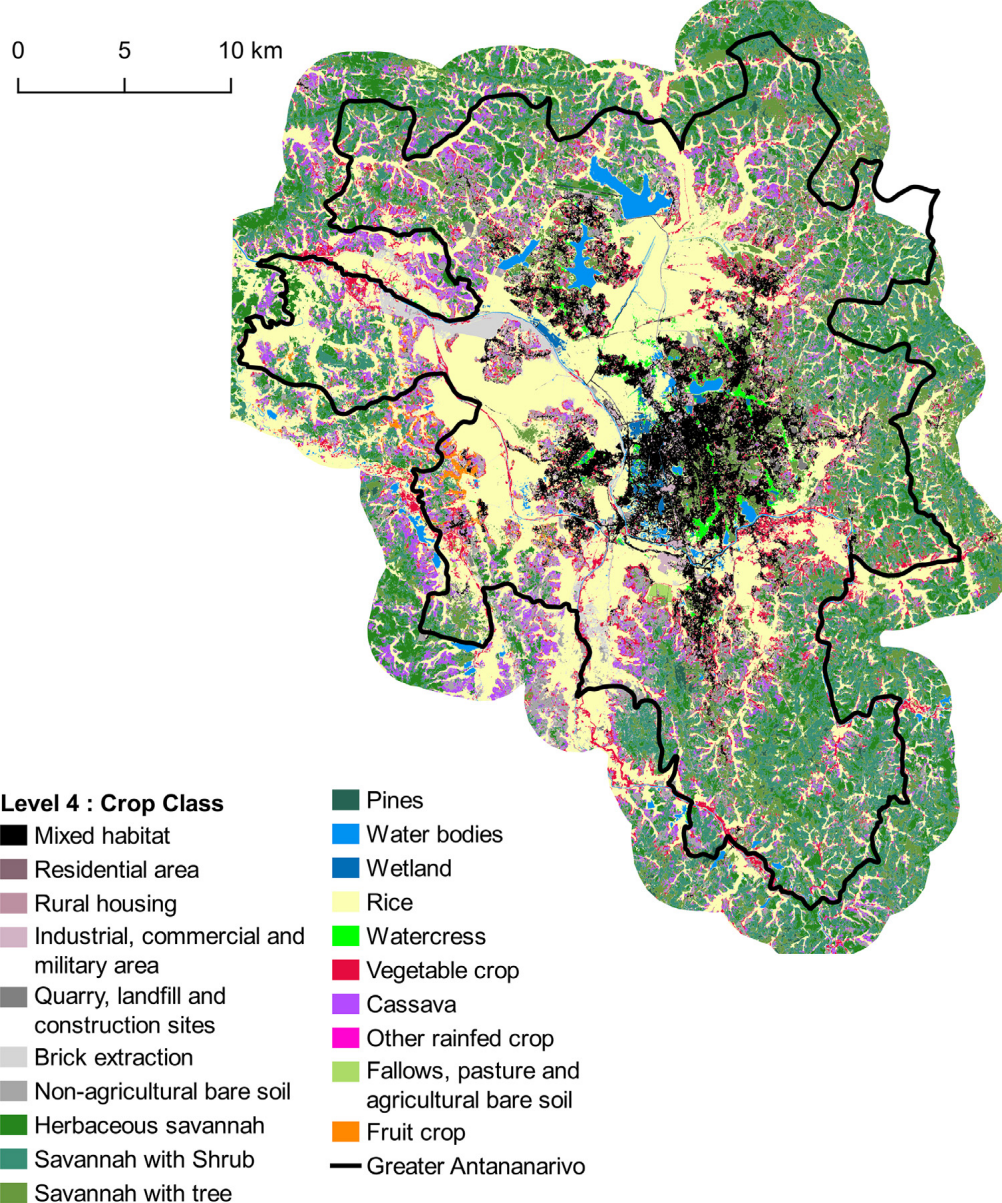
Land cover map corresponding to Level-3 with 20 classes. Vector file in ESRI shape format available here: http://dx.doi.org/10.18167/DVN1/NHE34C. This figure is a modified version of Fig. 5 published in this article [1].

Final maps in ESRI shapefile format are delivered in the local UTM projection (WGS 84 UTM 38 South, EPSG code 32,738). Data are referenced in the CIRAD Dataverse. Further description of these data and their use in a real world case study is detailed in the article [1].

## 2 Experimental design, materials, and methods

### 2.1 Materials

#### 2.1.1 2017 reference database and nomenclature

The reference database is organized according to a multi-level nomenclature (Cf. Table 1). Field surveys were performed during the end of the 2017 rainy season (March to April), which corresponds to the peak of the growing season. GPS waypoints were collected following an opportunistic sampling approach [2]. Waypoints were collected within the whole study area in order to have a representativeness of the existing types of crops and urban structures. GPS points have also been recorded for uncultivated and unbuilt plots such as savannah, forest or marsh. Each waypoint was then converted into a polygon by digitizing the boundaries of the corresponding land cover on a VHSR Pleiades image (0.5 m * 0.5 m pixel size). 981 additional polygons were digitized by photointerpretation of the Pleiades image for easily recognizable classes (housing, brick extraction, rice, watercress and savannah). The final ground database was thus composed of 3068 polygons (Cf. Fig. 1).

#### 2.1.2 Images

➢ **Very High Spatial Resolution (VHSR):**

Two 20×20 km Pleiades tiles (with spatial resolution of 2 m and 0.5 m) were acquired simultaneously on January 8, 2017, which corresponds to the middle of the rainy season in Madagascar. Pleiades images were acquired with the support of CNES (Centre National d'Etudes Spatiales: government agency responsible for shaping and implementing France's space policy in Europe). Pleiades images are not free and are available under condition of eligibility via the Theia consortium (Data and Services centre for continental surfaces) and the DINAMIS programme. More information is available on the Theia website (https://www.theia-land.fr/le-programme-isis-du-cnes-sintegre-a-dinamis).

➢ **High Spatial Resolution (HSR):**

The High Spatial Resolution time series consists of 50 images acquired between October 2016 and September 2017 (including 19 images from Landsat-8 and 62 images from Sentinel-2 (2 tiles and 31 acquisition dates)). Selection criteria for these images were: images should cover at least 20% of the study area and have less than 80% cloud cover per tile.

The Sentinel 2A and 2B satellites (S2A and S2B) have been deployed by the European Space Agency (ESA). The images offer 13 spectral bands with a spatial resolution between 10 m and 60 m. The interval between two subsequent acquisitions is 5 days considering both satellites. In this study, Sentinel-2 (S2) level-1C images provided by ESA were used and only 10 bands were kept with a resolution of 10 m and 20 m.

The Landsat-8 (L8) satellite was deployed by the National Aeronautics and Space Administration (NASA). The revisiting frequency is 16 days. L8 images have a spatial resolution of 15 m for the panchromatic band and 30 m for the multispectral bands.

The characteristics of the L8 and S2 images are different, but in tropical areas with high cloud cover, the combination of these sensors increases the probability of regularly observing the entire territory.

#### 2.1.3 Topography

The Shuttle Radar Topography Mission (SRTM) digital elevation model (DEM) at 30 m spatial resolution was downloaded from United States Geological Survey (USGS) website (https://earthexplorer.usgs.gov) to take into account the topography (altitude, slope) of the study zone.

### 2.2 Moringa processing chain to obtain land cover map in 2017

The Moringa processing chain was used to automate the production of land cover maps at Very High Spatial Resolution (VHSR) following a methodology that is particularly adapted to tropical agricultural systems (cloudy acquisitions, small field sizes, heterogeneous and fragmented landscapes) [3,4]. The Moringa chain can be downloaded at the following link: https://gitlab.irstea.fr/raffaele.gaetano/moringa

**Fig. 6:**
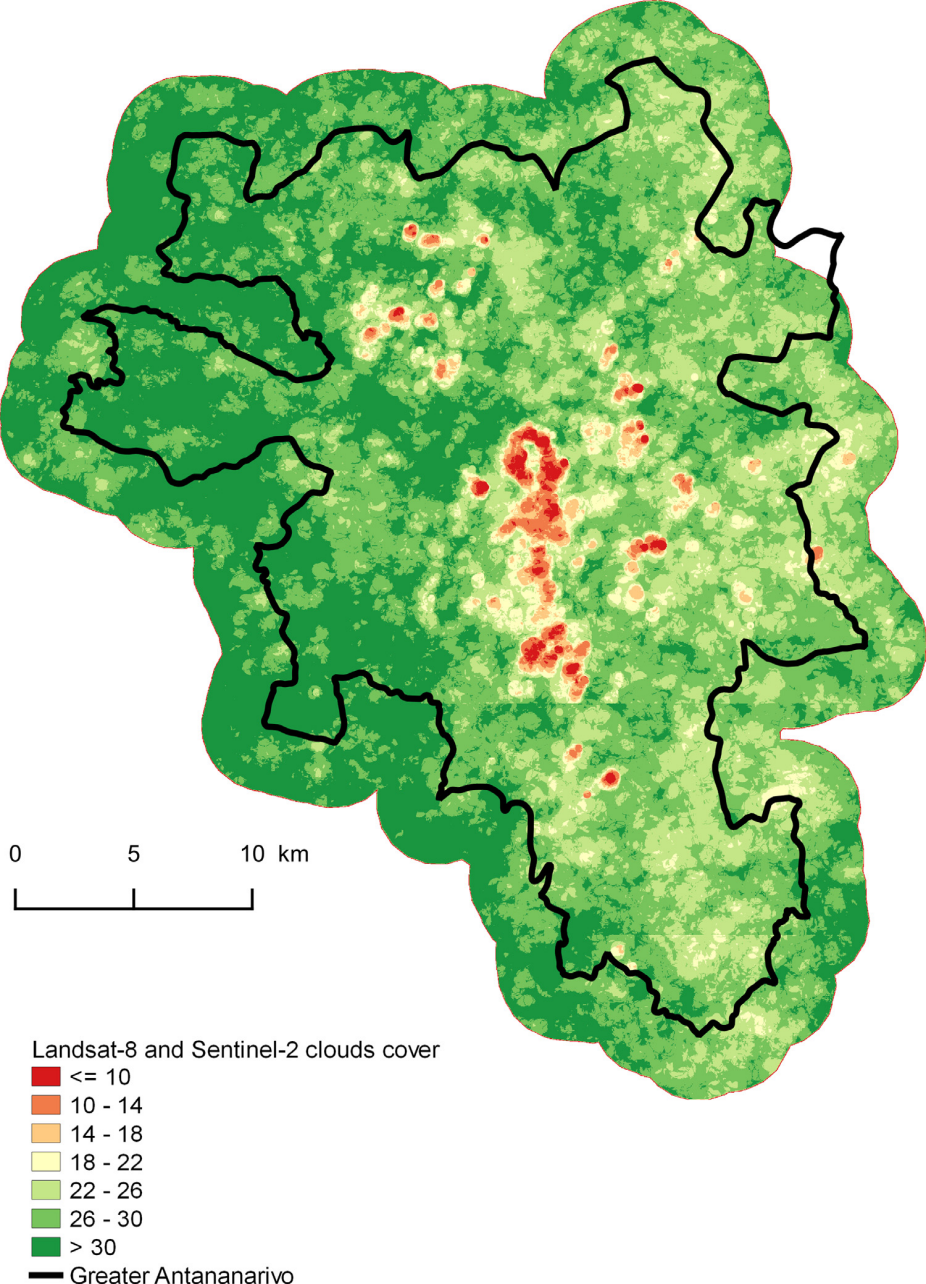
Number of images without clouds in the time serie (include Sentinel-2 and Landsat-8).

The methodology is based on the combined use of Very High Spatial Resolution (VHSR) Pleiades imagery, time series of Sentinel-2 and Landsat-8 High Spatial Resolution (HRS) optical images and a Digital Terrain Model (DTM) within an Object Based Image Analysis (OBIA) and Random Forest classification approach driven by a reference database combining in situ and photo-interpretation measurements. The chain is built upon the Orfeo Tool Box (OTB) applications, driven by python scripts. Some pre-processing steps are performed under QGiS. Main processes of the chain are summarized in Fig. 6. The following paragraphs describe specific parameters and useful elements of the classification method.

#### 2.2.1 VHSR pre-processing

Preprocessing steps were realized with Orfeo ToolBox [5] and consisted in Top of Atmosphere (TOA) reflectance calculation, and orthorectification of Pleiade image. Orthorectification of panchromatic and multispectral images was based on SRTM digital elevation model and the “Orthobase Madagascar” product (orthorectified mosaic of 2.5 m panchromatic SPOT5 images was acquired to serve as a reference for the co-registration of Pleiades images). These images are available under the condition of eligibility distributed by SEAS-OI (Survey of the Environment Assisted by Satellite in the Indian Ocean): http://www.seas-oi.fr. The two PAN and MS tiles were then mosaicked and the resulted mosaics were pansharpened using the Bayesian fusion algorithm of the OTB pansharpening module in order to obtain a multispectral mosaic at 0.5 m spatial resolution.

#### 2.2.2 HSR pre-processing

Pre-processing applied to HRS images is automated in the Moringa chain:

• The 62 Sentinel-2 tiles were mosaicked to produce a time series of 31 mosaics.
• For the Landsat-8 pansharpening processing is applied to bring the spatial resolution as close as possible to S2 images.

S2 and L8 images were coregistered to the VHSR Pleiades reference using an automatic procedure based on the homologous points extraction application of OTB. This processing is conceived to improve overlapping among the different remote sensing sources, and is crucial for the characterization of small scale objects.

Fmask tool [6] was used to produce the cloud masks corresponding to each image of the time series. The chain produces, from the cloud masks, an image illustrating the number of times a pixel is not covered by clouds in the time series. This illustration locates the areas where there is a risk of instability of the results on the maps if the number of clear acquisitions is low (Cf. Fig. 6).

#### 2.2.3 The variables used in the classification

➢ 6 common radiometric indices useful for land use characterization were chosen (Cf. Table 2)

**Table 2:** Description of variables extracted to compute the classification (with HSR = High Spatial Resolution and VHSR = Very High Spatial Resolution). This table is a modified version of Table 2 published in this article [1].

Type	HSR	VHRS	Topography
Spectral reflectance	Landsat-8: 7 bands and Sentinel-2: 10 bands		
Spectral indices	NDVI^1^[7], MNDVI^2^[8], NDWI^3^[9], MNDWI^4^[10], brightness index^5^ and RNDVI^6^[11]		
Textural indices		Energy, Contrast et Variance Haralick indices [12] calculated at 2 windows size: 5 × 5 and 11 × 11	
Topographic indices			Altitude and slope
**number**	**1024**	**6**	**2**

1: Normalized Difference Vegetation Index. 2: Modified Normalized Difference Vegetation Index. 3: Normalized Difference Water Index. 4: Modified Normalized Difference Water Index. 5: Square root of the sum of squared values of all bands. 6: Rededge NDVI (only for Sentinel-2).
➢ Textures are important to detect visible patterns on the THRS image such as tree alignments in agricultural crops. In the Moringa chain, these texture indices are the only variables derived from the VHSR image. The OTB "Haralick Texture Extraction" algorithm was used and applied to the panchromatic image (Cf. Table 2)
➢ Slopes were calculated using QGIS software. DTM and slopes are used as variables in the classification process.

#### 2.2.4 Object based classification

The Moringa processing chain is designed to provide object-based supervised classification, and operates by first performing the segmentation of the VHSR image to generate a suitable object layer. The method described in [13], implemented in the large scale version of OTB's GenericRegionMerging application [14], was used to perform the segmentation. To obtain a segmentation result adapted to our study, parameters for the homogeneity criteria and the maximum heterogeneity threshold were assessed using a grid search on several representative subsets of the VHSR pansharpened image. The following parameters were finally chosen:

➢ Scale parameter: 150
➢ Shape parameter: 0.3
➢ Compactness parameter: 0.7

Training samples were subsequently generated by intersecting the so-obtained segmentation with the reference polygons available in the GIS dataset, and attributed using the spatial means over every band and index listed in Table 2. Random Forest (RF) classification algorithm [14,15] was chosen for classification considering its robustness when working with heterogeneous data, such as in our study (data from several sensors combined with altitude, slopes and textural indices).

An independent RF model was built for each nomenclature level, and applied for the classification of the whole set of objects, which were beforehand attributed in the same way described for the training samples. At the end of the process, the four land use maps are made available in vector and raster format.

#### 2.2.5 Validation of 2017 maps

We here use the k-fold cross-validation technique to evaluate the accuracy of the provided land use maps. The specific validation protocol (number of folds, accuracy metrics) for these maps is the same already used in [1,4]

These quality indicators (global accuracy, Kappa, fscore) are given in Table 3.

**Table 3:** Global and class accuracy indices by level. This table is a modified version of Table 4 published in this article [1].

LEVEL 1	F1-SCORE	LEVEL 2	F1-SCORE	LEVEL 3	F1-SCORE	LEVEL 4	F1-SCORE
Non crop	96.13%	Urban area	91.6%	Built-up surface	83.5%	Mixed habitat	65.1%
						Residential area	8.2%
						Rural housing	52.8%
				Industrial, commercial and military area	81.8%	Industrial, commercial and military area	84.1%
				Quarry, landfill and construction site	86.8%	Quarry, landfill and construction site	68.0%
						Brick extraction	94.7%
		Natural spaces	73.1%	Bare non-agricultural soil	41.8%	Bare non-agricultural soil	44.9%
				Savannah	75.6%	Herbaceous savannah	62.4%
						Shrub savannah	58.4%
		Forest	86.8%	Forest	86.9%	Tree savannah	63.0%
						Pines	63.0%
		Waterbodies and wetland	90.4%	Water bodies	97.5%	Water bodies	97.4%
				Wetland	61.9%	Wetland	70.2%
Crop	91.7%	Annual and pluriannual crop	88.5%	Irrigated crop	89.1%	Rice	88.0%
						Watercress	78.3%
				Vegetable crop	37.1%	Vegetable crop	41.1%
				Rainfed crop	47.4%	Cassava	48.6%
						Other rainfed crop	0%
		Fallows, pasture and bare agricultural soil	63.7%	Fallows, pasture and bare agricultural soil	66.8%	Fallows, pasture and bare agricultural soil	67.7%
		Fruit crop	45.0%	Fruit crop	68.%	Fruit crop	67.7%
**Overall accuracy**	**94.78%**		**86.83%**		**84.08%**		**76.56%**
**Kappa index**	**0.88**		**0.83**		**0.81**		**0.74**

#### 2.2.6 Smoothing by majority filter

A majority filter was applied to the rasterized classification to smooth out contours and remove isolated pixels. OTB's Classification Map Regularization tool was used. The size of the structuring element can be adjusted to measure the intensity of the smoothing. To limit the degradation of the classification, a filter of radius 1, corresponding to a 3 × 3 pixel window, was chosen.

## Declaration of Competing Interest

The authors declare that they have no knowledge of competing financial interests or personal relationships which have, or could be perceived to have, influenced the work reported in this article.

## Appendix Supplementary materials

Image, application 1
